# Curcumol may alleviate psoriasis-like inflammation by inhibiting keratinocyte proliferation and inflammatory gene expression via JAK1/STAT3 signaling

**DOI:** 10.18632/aging.203287

**Published:** 2021-07-27

**Authors:** Mingfen Lv, Junyi Shao, Fan Jiang, Jingjing Liu

**Affiliations:** 1 Department of Dermatology, The First Affiliated Hospital of Wenzhou Medical University, Wenzhou 325000, Zhejiang, P.R. China

**Keywords:** psoriasis, curcumol, proliferation, inflammation, differentiation

## Abstract

Psoriasis is a chronic inflammatory skin disease characterized by abnormal proliferation and differentiation of keratinocytes. Since curcumol exhibits anti-inflammatory properties in various diseases, we investigated its anti-inflammatory potential in stimulated human keratinocytes. Our data show that curcumol significantly inhibits proliferation and induces cell cycle arrest in NHEK cells stimulated with proinflammatory cytokines (IL-1α, IL-17A, IL-22, oncostatin M, and TNF-α; mix M5). In addition, curcumol markedly ameliorates inflammatory response and promotes differentiation of M5-stimulated NHEK cells. Curcumol inhibits activity of JAK1, resulting in the inhibition of STAT3, downregulation of cyclin D2, and cell cycle arrest in stimulated NHEK cells. Together, our data show that curcumol reduces proliferation and inflammatory gene expression in stimulated keratinocytes by inhibiting the JAK1/STAT3 signaling, suggesting that it might serve as a potential therapeutic option for the treatment of psoriasis.

## INTRODUCTION

Psoriasis is a chronic, systemic inflammatory skin condition that affects nearly 3% of the world’s population [1, 2]. The clinical manifestations of psoriasis are erythema and scaly skin, which can affect the entire body [3]. Psoriasis outbreaks are triggered by multiple factors, including hereditary factors, external environmental factors, upper respiratory tract infections, and external living conditions [4]. Psoriasis can seriously affect patient's quality of life; 20-30% of patients with psoriasis have joint pains [5]. In addition, patients with moderate or severe psoriasis are at an increased risk for metabolic syndrome and atherosclerotic cardiovascular disease [6]. Although current treatment measures for psoriasis are effective, a long-term remission cannot be achieved [7]. Therefore, it is necessary to develop novel therapeutic approaches for the treatment of psoriasis.

Curcumol is a polyphenol compound isolated from the rhizome of *Rhizoma Curcumae*; it is a pharmacological sesquiterpene with anti-tumor, anti-inflammatory, anti-viral, and anti-bacterial effects [8]. Curcumol inhibits proliferation of gastric cancer cells [9]. In addition, Li et al. indicated that curcumol reduces cigarette smoke extract-induced inflammatory injury in macrophages via reducing the release of intracellular reactive oxygen species (ROS) and pro-inflammatory factors [10]. Moreover, Chen et al. found that curcumol inhibits LPS-induced inflammatory response in RAW264.7 cells via suppressing the production of inflammatory cytokines, including TNF-α, IL-1β and IL-6 [11]. However, the biological role of curcumol in psoriasis remains unclear. Thus, it is urgent to investigate the role of curcumol in psoriasis.

The JAK/STAT signaling pathway is a cytokine stimulated signal transduction pathway that has been discovered in recent years. The transmission process of this signaling pathway is mainly composed of three components: tyrosine kinase associated receptor, Janus kinase and signal transducer and activator of transcription [12]. This pathway is involved in many important biological processes such as cell proliferation, differentiation, apoptosis, and immune regulation [13]. Activation of the JAK/STAT signal transduction pathway promotes cell growth and cell cycle transformation [14]. It's reported that matrine could inhibit the activation of JAK/STAT pathway to increase the inhibitory effect of drugs on lung cancer cells [15]. However, the mechanism by which curcumol regulates the occurrence and development of psoriasis remains unclear. This study aimed to investigate the role of curcumol during the progression of psoriasis.

M5 ((IL-1α, IL-17A, IL-22, oncostatin M, and TNF-α) can induce keratinocytes to exhibit some of the characteristics of psoriatic keratinocytes *in vitro* [16]. Meanwhile, it has been reported that IL-17 and IL-22 enhance cutaneous inflammation, leading to epidermal thickening of psoriatic lesions and adherence of psoriatic scales [17]. Therefore, in the present study, Normal human epidermal keratinocytes (NHEK) cells were treated with M5 to establish the model of psoriatic keratinocyte *in vitro*.

## RESULTS

### Curcumol inhibits proliferation and cell cycle progression and promote apoptosis in stimulated human keratinocytes

**Figure 1:**
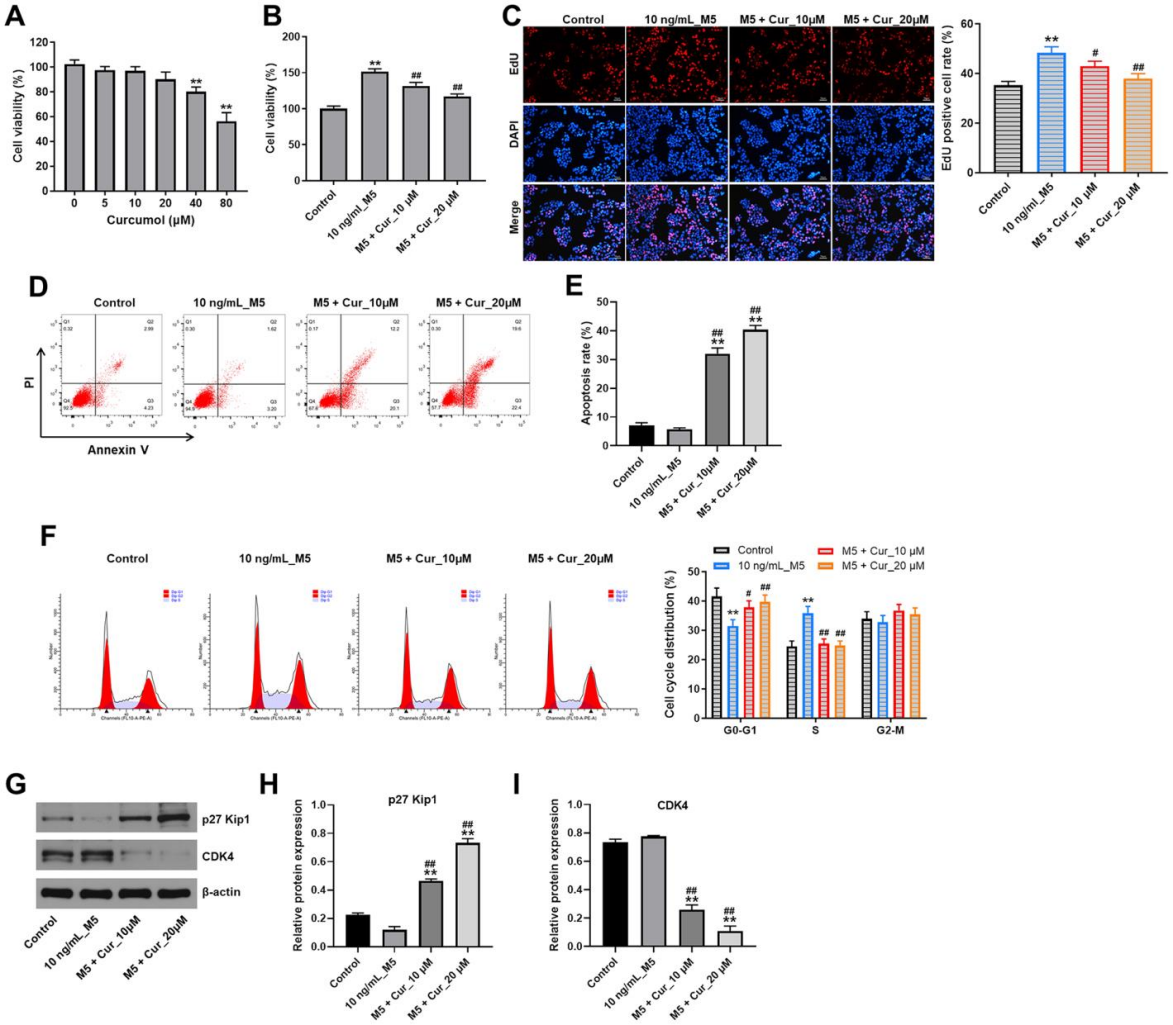
**Curcumol inhibits proliferation and cell cycle progression and promote apoptosis in stimulated human keratinocytes.** (**A**) CCK-8 cell viability assay in NHEK cells treated with curcumol (0, 5, 10, 20, 40 or 80 μM) for 24 h. (**B**) CCK-8 cell viability assay in NHEK cells pre-incubated with curcumol (10 or 20 μM) for 24 h, and stimulated with 10 ng/mL of M5 mix for 24 h. (**C**) Relative fluorescence levels were quantified by EdU and DAPI staining. (**D**, **E**) Flow cytometer assay was used to analyze cell apoptosis. (**F**) Cell cycle distribution was measured by flow cytometry. (**G**–**I**) Western blot was performed to measure the expressions of p27 Kip1 and CDK4 in NHEK cells. **P<0.01 compared with control group; ^#^P<0.05, ^##^P<0.01 compared with 10 ng/mL_M5 group.

First, we assessed the effect of curcumol on the viability of normal human epidermal keratinocyte (NHEK) cells using CCK-8 assay. As shown in Figure 1A, 24 h treatment with 5, 10, and 20 μM curcumol had a very limited effect on NHEK cell viability; however, 40 and 80 μM curcumol significantly reduced the cell viability. Thus, we used 10 and 20 μM curcumol in the following experiments. We then investigated the effect of curcumol on viability and proliferation of NHEK cells stimulated with a combination of proinflammatory cytokines characteristic for psoriasis (IL-1α, IL-17A, IL-22, oncostatin M, and TNF-α; mix M5). As shown in Figure 1B, 1C, M5 markedly increased the viability and proliferation of NHEK cells; however, these effects were reversed by curcumol. In addition, the result of apoptosis evaluation showed that M5 decreased the apoptosis of NHEK cells, while this phenomenon was notably reversed by curcumol (Figure 1D, 1E). Moreover, M5 notably decreased the percentage of NHEK cells in G0/G1 phases, but increased the percentage of cells in S-phase; however, these effects were reversed by curcumol treatment (Figure 1F). Furthermore, the expressions of cell cycle-associated proteins p27 Kip1 and CDK4 were determined using western blot assay. Consistently, M5 downregulated the level of p27 Kip1 and upregulated the level of CDK4; however, the effects of M5 on these proteins were all reversed by curcumol (Figure 1G–1I). All in all, these data suggested that curcumol could inhibit proliferation and cell cycle progression and promote apoptosis of M5-stimulated NHEK cells.

### Curcumol suppresses inflammatory gene expression in M5-stimulated NHEK cells

**Figure 2:**
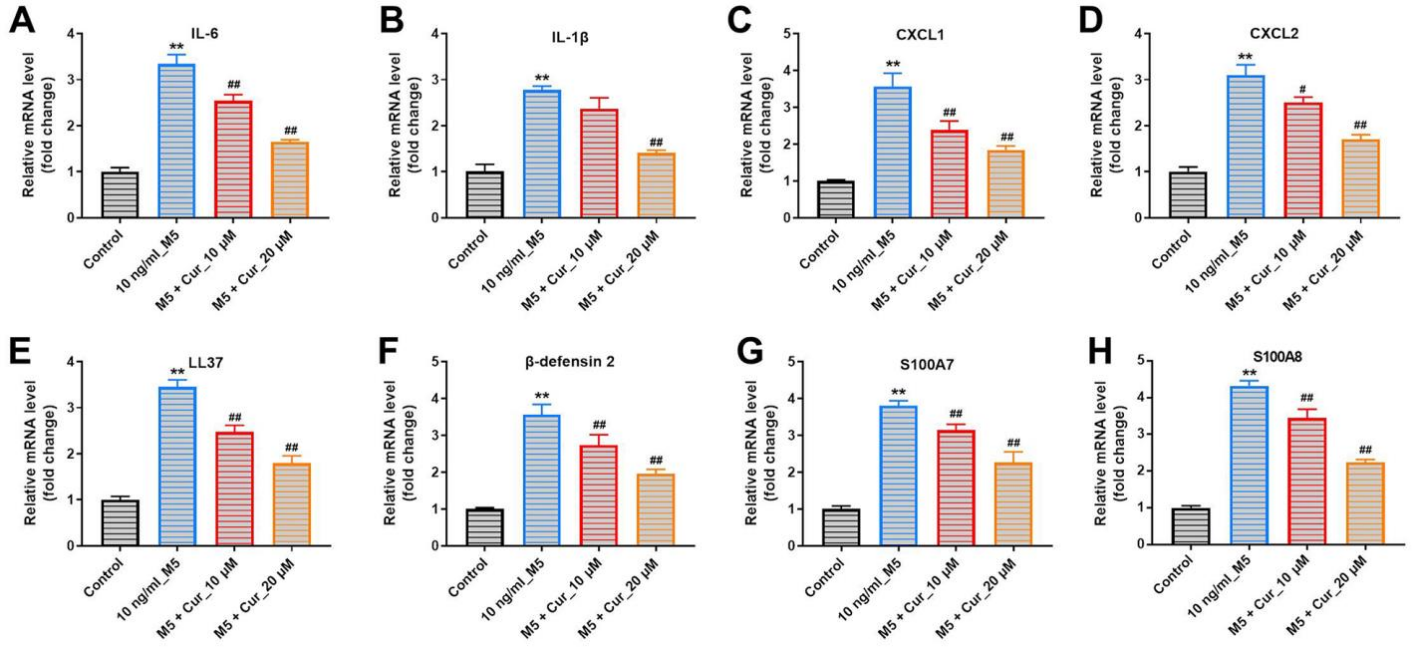
**Curcumol suppresses inflammatory gene expression in M5-treated NHEK cells.** NHEK cells were preincubated with curcumol (10 or 20 μM) for 24 h, stimulated with M5 (10 ng/mL) for 24 h, and gene expression of IL-6 (**A**), IL-1β (**B**), CXCL1 (**C**), CXCL2 (**D**), LL37 (**E**), β-defensin-2 (**F**), S100A7 (**G**), and S100A8 (**H**) was analyzed by RT-qPCR. **P<0.01 compared with control group; ^#^P<0.05, ^##^P<0.01 compared with 10 ng/mL_M5 group.

Keratinocyte exposure to M5-proinflammatory cytokines induces expression of proinflammatory mediators, including cytokines, chemokines, and antimicrobial peptides [18]. Thus, we investigated whether curcumol could attenuate the inflammatory gene expression in M5-stimulated NHEK cells. As shown in Figure 2, M5 exposure significantly increased mRNA levels of cytokines (IL-6, IL-1β), chemokines (CXCL1, CXCL2), and antimicrobial peptides (LL37, β-defensin-2, S100A7 and S100A8) in NHEK cells. Importantly, this M5-induced inflammatory gene expression was notably suppressed by curcumol treatment, indicating that curcumol attenuates the inflammatory response in M5-stimulated NHEK cells.

### Curcumol attenuates M5-induced oxidative stress damage in NHEK cells

**Figure 3:**
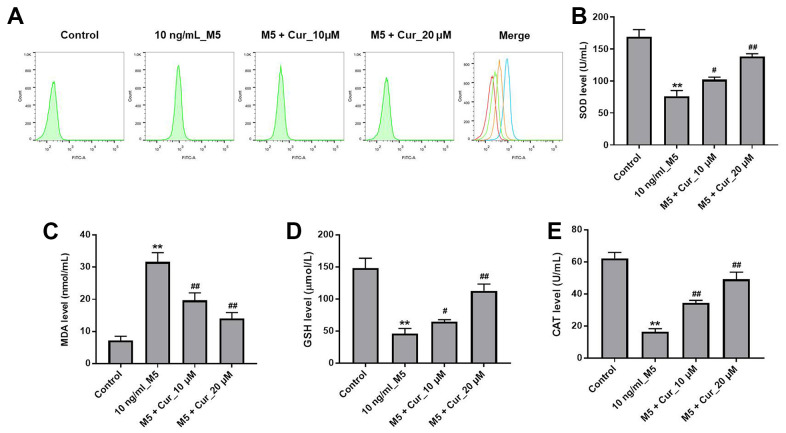
**Curcumol attenuates M5-induced oxidative stress damage in NHEK cells.** NHEK cells were treated with curcumol (10 or 20 μM) for 24 h, and then stimulated with M5 (10 ng/mL) for 24 h. (**A**) ROS production in NHEK cells was detected by flow cytometry. (**B**–**E**) ELISA was used to detect the levels of SOD (**B**), MDA (**C**), GSH (**D**), and CAT (**E**) in the supernatants of NHEK cells. **P<0.01 compared with control group; ^#^P<0.05, ^##^P<0.01 compared with 10 ng/mL_M5 group.

We further investigated whether curcumol could protect NHEK cells against M5-induced oxidative stress damage. As illustrated in Figure 3A, curcumol dramatically inhibited M5-induced ROS production in NHEK cells. In addition, M5 significantly reduced the production of superoxide dismutase (SOD), glutathione (GSH), and catalase (CAT), and increased the production of malondialdehyde (MDA) in NHEK cells. However, these changes were markedly reversed by curcumol (Figure 3B–3E). These data indicated that curcumol could attenuate the M5-induced oxidative stress damage in NHEK cells.

### Curcumol promotes differentiation of M5-stimulated NHEK cells

**Figure 4:**
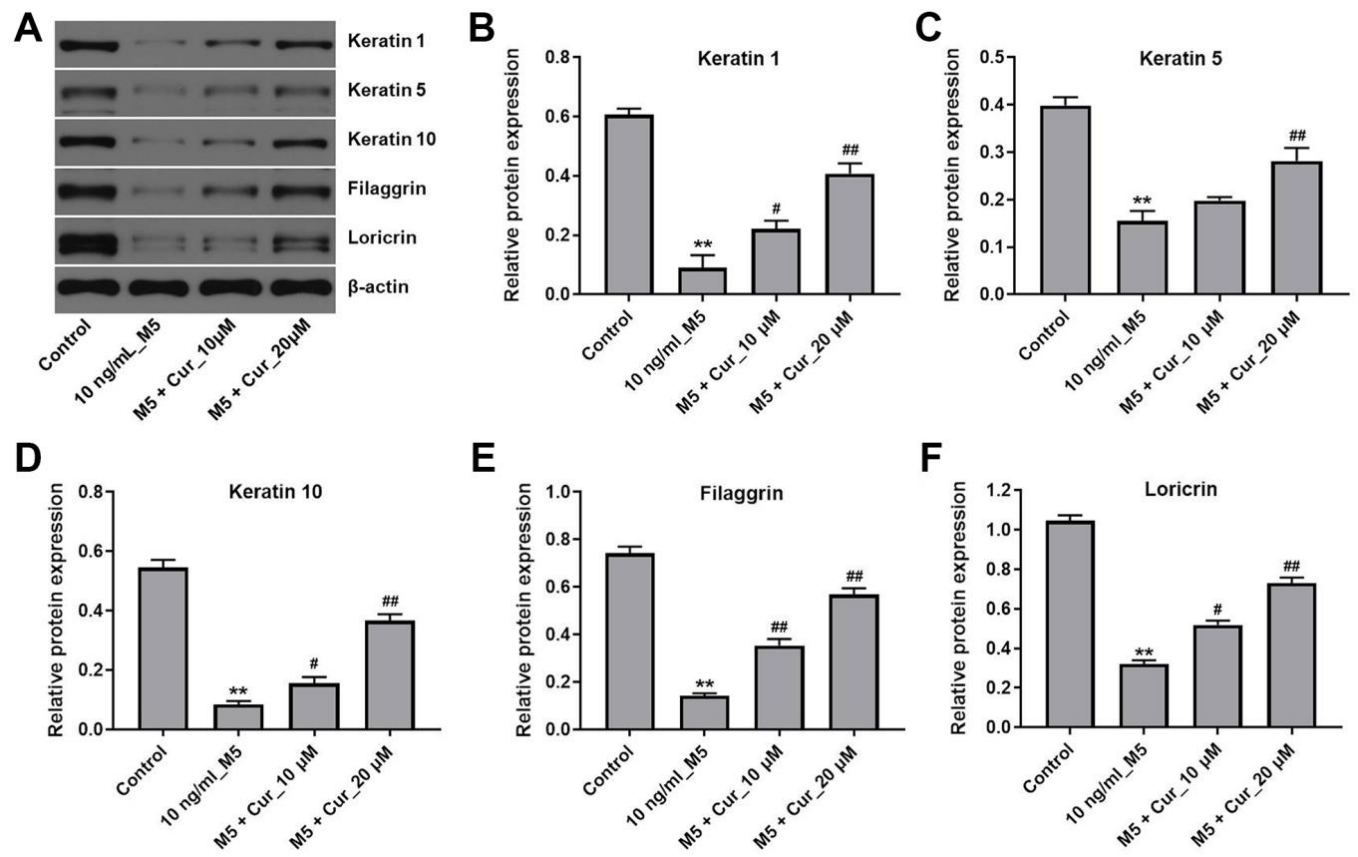
**Curcumol promotes differentiation in M5-stimulated NHEK cells.** NHEK cells were treated with curcumol (10 or 20 μM) for 24 h, and then stimulated with M5 (10 ng/mL) for 24 h. (**A**) Expression of keratin 1, keratin 5, keratin 10, filaggrin and loricrin in NHEK cells was analyzed by western blotting. (**B**–**F**) The relative expression of keratin 1 (**B**), keratin 5 (**C**), keratin 10 (**D**), filaggrin (**E**), and loricrin (**F**) quantified via normalization to β-actin. **P<0.01 compared with control group; ^#^P<0.05, ^##^P<0.01 compared with 10 ng/mL_M5 group.

Psoriasis is characterized by the downregulation of keratinocyte differentiation markers [19]. Thus, we investigated whether curcumol could promote keratinocyte differentiation in M5-treated NHEK cells. As shown in Figure 4, curcumol reversed changes in protein levels of the differentiation markers keratin 1, keratin 5, keratin 10, filaggrin, and loricrin that were induced by M5 treatment in NHEK cells. These results indicate that curcumol promotes differentiation of M5-stimulated NHEK cells.

### Curcumol inhibits proliferation of M5-stimulated NHEK cells via downregulation of p-STAT3/cyclin D2 axis

**Figure 5:**
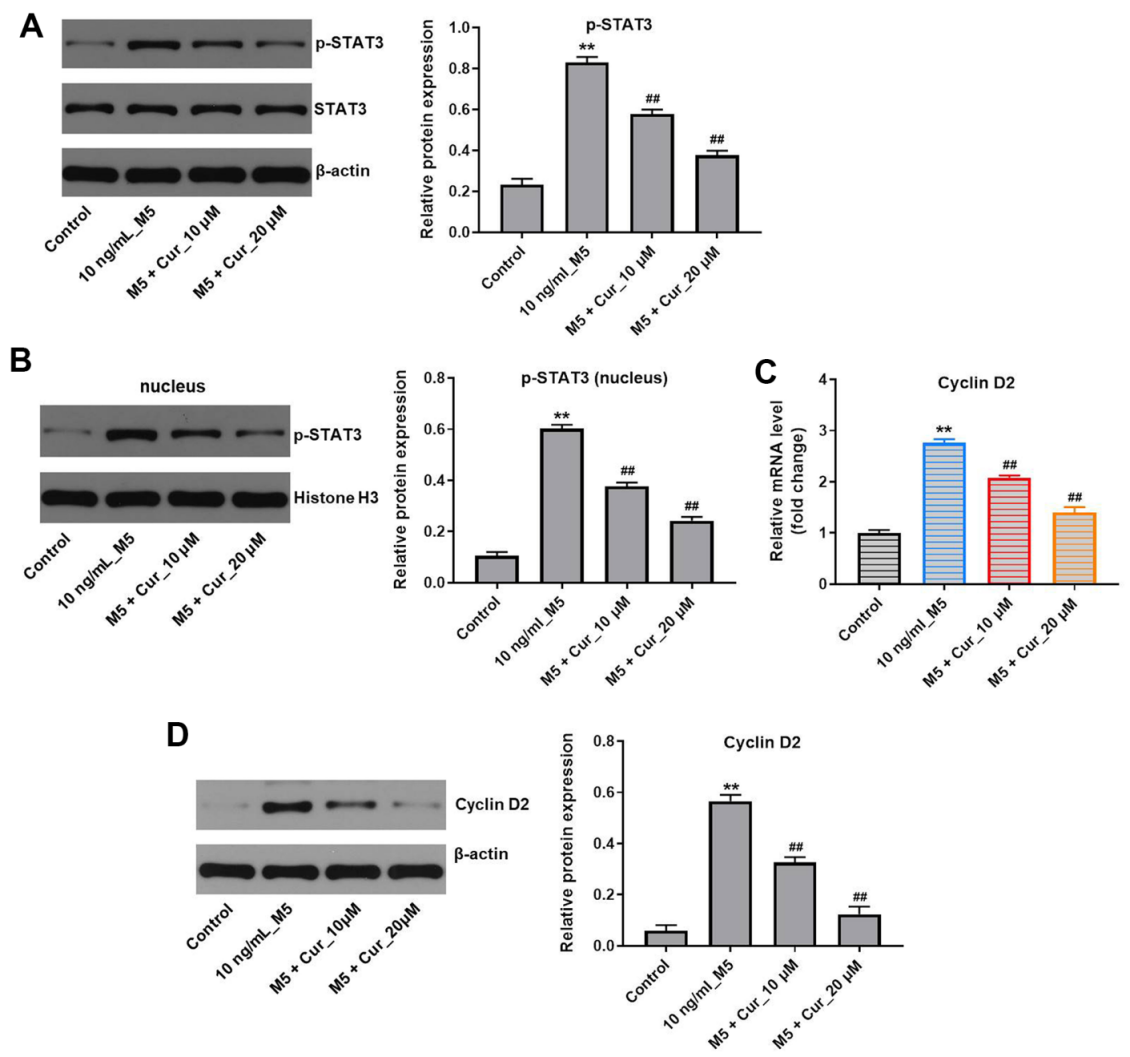
**Curcumol inhibits proliferation of M5-treated NHEK cells via downregulation of p-STAT3/cyclin D2 axis.** NHEK cells were treated with curcumol (10 or 20 μM) for 24 h, and then stimulated with M5 (10 ng/mL) for 24 h. (**A**) The expression level of p-STAT3 in NHEK cells was detected by western blotting, and normalized to STAT3. (**B**) The nuclear levels of p-STAT3 were analyzed by western blotting, and normalized to Histone H3. (**C**) Cyclin D2 mRNA levels were analyzed by RT-qPCR. (**D**) Cyclin D2 protein levels were analyzed by western blotting, and normalized to β-actin. **P<0.01 compared with control group; ^##^P<0.01 compared with 10 ng/mL_M5 group.

Since activation of STAT3 plays a critical role in psoriasis [20], we investigated the effect of curcumol on STAT3 signaling in M5-stimulated NHEK cells. As shown in Figure 5A, curcumol markedly decreased the level of p-STAT3 (phospho Y705) in M5-stimulated NHEK cells (Figure 5A). As the activated STAT3 translocates from cytoplasm to the nucleus, where it induces transcription of target genes, including cyclin D2 [21, 22], we also analyzed the levels of nuclear STAT3 after curcumol treatment. In addition, since Belsõ et al. found that the expression of cyclin D2 mRNA in psoriatic epidermis cells was significantly higher than that in normal epidermis cells [23]; thus, cyclin D2 was investigated as well. As shown in Figure 5B, curcumol reduced the nuclear STAT3 levels in M5-stimulated NHEK cells. Moreover, curcumol notably downregulated the mRNA and protein levels of cyclin D2 in M5-treated NHEK cells (Figure 5C, 5D), suggesting that curcumol inhibits proliferation of M5-treated NHEK cells via downregulation of the STAT3/cyclin D2 axis.

### Curcumol inhibits proliferation of M5-stimulated NHEK cells via downregulation of JAK1/STAT3 signaling pathway

**Figure 6:**
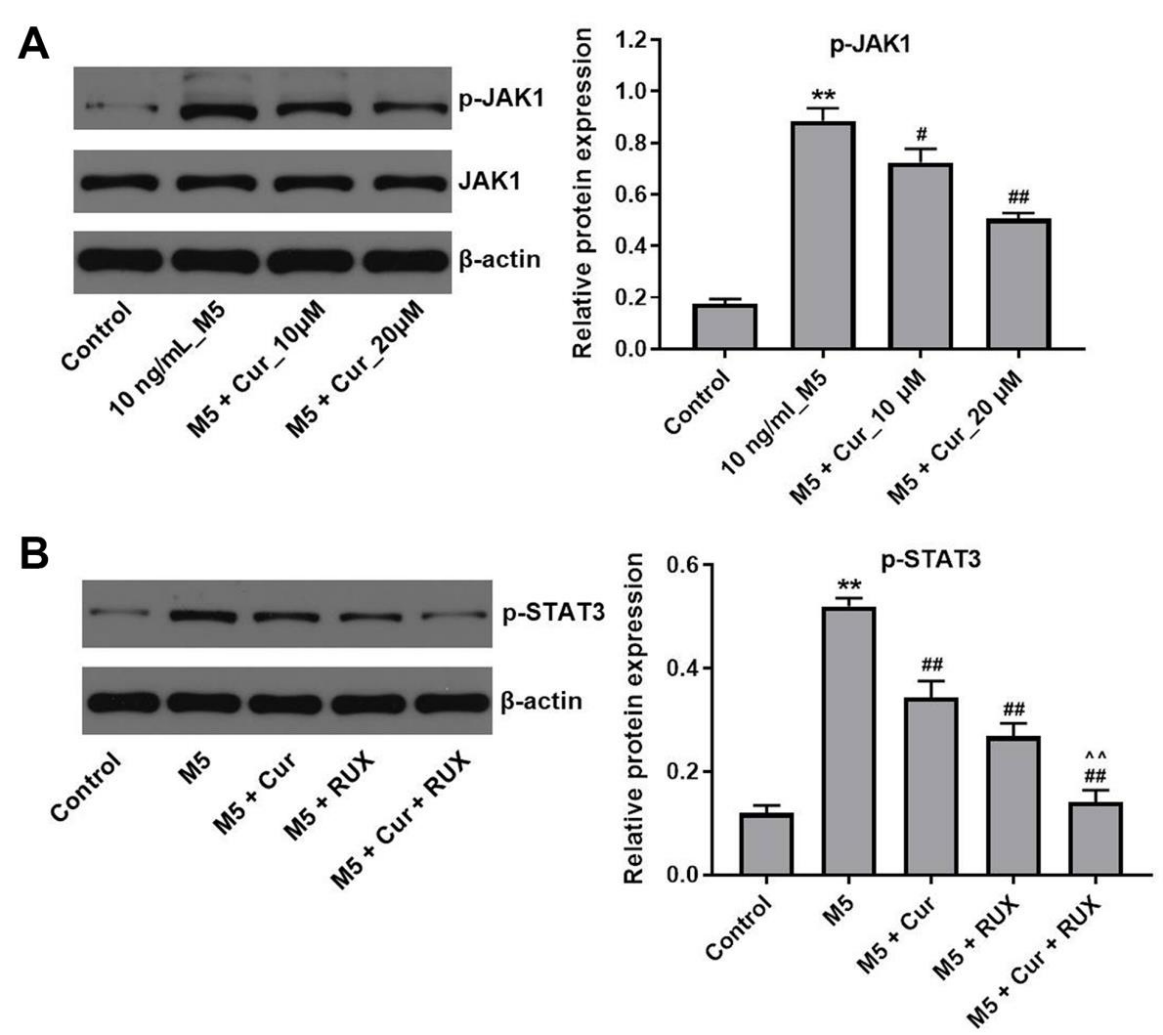
**Curcumol inhibits proliferation of M5-treated NHEK cells via downregulation of JAK1/STAT3 signaling.** (**A**) NHEK cells were treated with curcumol (10 or 20 μM; 24 h), and stimulated with M5 (10 ng/mL; 24 h). Levels of p-JAK1 were analyzed by western blotting, and normalized to JAK1. (**B**) NHEK cells were treated with 20 μM curcumol or/and 3μM ruxolitinib (RUX) for 24 h, and then stimulated with M5 (10 ng/mL) for 24 h. Levels of p-STAT3 were analyzed by western blotting, and normalized to STAT3. **P<0.01 compared with control group; ^#^P<0.05, ^##^P<0.01 compared with M5 group; ^^P<0.01 compared with M5 + RUX group.

Since STAT3 activation is mediated by the Janus kinases (JAKs) [24], we investigated whether curcumol affects the JAK activity in M5-stimulated NHEK cells. As shown in Figure 6A, curcumol markedly decreased the phosphorylation level of JAK1 in M5-treated NHEK cells. In addition, a preincubation with the JAK1/2 inhibitor ruxolitinib reduced the phosphorylation levels of STAT3 in M5-stimulated NHEK cells, and this effect was further enhanced by curcumol (Figure 6B). Together, these data indicate that curcumol inhibits proliferation of stimulated human keratinocytes by suppressing the JAK1/STAT3 signaling pathway.

## DISCUSSION

The pathogenesis of psoriasis is extremely complex and involves numerous immune and inflammatory mediators, including IL-1α, TNF-α, IL-17A, and IL-22 [18, 25]. Thus, these cytokines are often used as *in vitro* inducers to stimulate keratinocyte hyperproliferation and inflammation [18]. In this study, a combination of IL-1α, IL-17A, IL-22, oncostatin M, and TNF-α (mix M5) was used to trigger psoriasis-like changes in NHEK cells *in vitro*. Our data demonstrated that M5 markedly promoted proliferation and cell cycle progression in NHEK cells. Importantly, curcumol significantly inhibited the M5-induced proliferation and cell cycle progression of NHEK cells. Sun et al. found that berberine inhibited proliferation of IL-22-stimulated keratinocytes [26]. This is consistent with our data that suggest that curcumol may alleviate psoriasis-like inflammation in keratinocytes by inhibiting their proliferation.

Previous studies have shown that M5 activates keratinocytes, leading to the release of cytokines, chemokines and antimicrobial peptides [27]. Our data demonstrated that M5 notably upregulated mRNA levels of cytokines (IL-6, IL-1β), chemokines (CXCL1, CXCL2), and antimicrobial peptides (LL37, β-defensin-2, S100A7 and S100A8) in NHEK cells. However, curcumol remarkably attenuated the expression of these inflammatory mediators in M5-treated NHEK cells. Liu et al. found that cimifugin inhibited TNFα-induced inflammatory response in NHEK cells [28]. In addition, betulinic acid attenuated psoriatic skin inflammation in a murine model of imiquimod-induced psoriasis via downregulation of pro-inflammatory mediators IL-6 and TNFα [29]. These findings indicate that curcumol might have a protective effect against M5-induced inflammatory injury in keratinocytes.

Previous studies have indicated an important role of STAT3 in the development and pathogenesis of psoriasis [30, 31]. IL-6 and IL-22 can induce STAT3 activation, leading to keratinocyte hyperproliferation and excessive inflammatory response [32, 33]. In addition, activation of STAT3 induces expression of cyclin D2, thereby promoting cell cycle progression [34]. Here, we found that curcumol reduced STAT3 phosphorylation and cyclin D2 expression in M5-stimulated NHEK cells, indicating that curcumol might block the STAT3-cyclin D2 signaling in keratinocytes. Curcumol was shown to suppress the activation of STAT3 in fibroblast-like synoviocytes in patients with rheumatoid arthritis [35]. However, the mechanisms remain largely unknown. Since JAKs are the upstream activators of STATs [36], inhibition of JAKs may block the activation of STAT3 in M5-stimulated NHEK cells. In this study, we found that curcumol decreased the phosphorylation level of JAK1 in M5-treated NHEK cells. In addition, curcumol enhanced the inhibitory effect of JAK1/2 inhibitor ruxolitinib on STAT3 activity, indicating that curcumol inhibits the STAT3 activation by inhibiting JAK1. Zuo et al. found that curcumol reduced the levels of phosphorylated STAT3 in hepatic cancer cells via JAK1 and JAK2 pathways [37]; this is in line with our results. Taken together, our results suggest that curcumol inhibits proliferation and inflammatory response in M5-stimulated NHEK cells via inhibition of the JAK1/STAT3/cyclin D2 pathway.

Obliviously, the present study contains certain limitations. For example, the detailed mechanism by which Curcumol regulates JAK1/STAT3 signaling pathway remains unclear; other signaling pathways associated with JAK1/STAT3 signaling pathway not been further explored. Therefore, additional studies are required in the future.

In summary, our results show that curcumol reduces proliferation and inflammatory response in keratinocytes stimulated with proinflammatory cytokines by inhibiting the JAK1/STAT3 pathway, suggesting that curcumol might provide a potential therapeutic option for the treatment of psoriasis.

## MATERIALS AND METHODS

### Cell culture

Normal human epidermal keratinocytes (NHEK) cell line was purchased from ScienCell Research Laboratories (Carlsbad, CA, USA). Cells were cultured in Dulbecco's Modified Eagle Medium (DMEM, Thermo Fisher Scientific, Waltham, MA, USA) containing 10% fetal bovine serum (FBS, Thermo Fisher Scientific) and 100 U/ml penicillin and streptomycin (Thermo Fisher Scientific) at 37° C in an incubator with 5% CO_2_.

### Cell counting kit-8 (CCK-8) assay

CCK-8 assay was performed to measure viability of NHEK cells. NHEK cells (5 x 10^3^ cells/well) were seeded into a 96-well plate, and incubated overnight at 37° C. NHEK cells were pre-treated with 10 or 20 μM curcumol (MedChemExpress, Monmouth Junction, NJ, USA) for 24 h, and then exposed to 10 ng/mL mixture of five proinflammatory cytokines (M5; Sigma-Aldrich, St. Louis, MO, USA) for another 24 h. After that, 10 μL CCK-8 (Dojindo, Kumamoto, Japan) reagent was added into each well, and cells were incubated for another 2 h at 37° C. The absorbance of each well at 450 nm was measured using a microplate reader (Bio-Rad, Hercules, CA, USA).

### 5-Ethynyl-20-deoxyuridine (EdU) assay

EdU assay was performed using the EdU DNA Proliferation *in vitro* Detection kit (Ribobio Biology, Guangzhou, China). NHEK cells (4×10^5^ cells/well) were placed into 24-well plate and incubated at 37° C overnight. Cells were then incubated with 50 μM EdU for 2 h at 37° C, stained with Apollo staining solution for 30 min at 37° C in dark, and observed under a fluorescence microscope.

### Flow cytometry assay

Cells were collected, resuspended in PBS, and fixed overnight in 70% pre-cooled ethanol at 4° C. After that, fixed cells (1×10^6^ cells/mL) were washed twice with PBS and stained with PI/RNase Staining Buffer (BD Biosciences, Franklin Lakes, NJ, USA) in the dark for 30 min at 37° C. The cell cycle distribution was analyzed using the FACScan™ flow cytometer (BD Biosciences).

### Evaluation of cell apoptosis

NHEK cells were placed into 6-well plates as density of 3 × 105 cell/well. Then, Annexin V-FITC Apoptosis Detection Kit (Sungene Biotech, Tianjin, China) was used to detect the apoptosis of NHEK cells. NHEK cells were incubated with Annexin V-FITC (5 μl) and PI (5 μl) at 4° C for 15 min. Next, a BD AriaIII flow cytometry system (BD Biosciences, Franklin Lake, NJ, USA) was used to evaluate cell apoptosis.

### Western blot assay

The BCA protein assay kit (Aspen Biotechnology, Wuhan, China) was used to determine protein concentration. Equal protein amounts (30 μg/lane) were separated by 10% SDS-PAGE, and transferred onto PVDF membranes (Millipore, Billerica, MA, USA). The membranes were blocked in 5% skimmed milk for 1 h at room temperature, and incubated at 4° C overnight with the following primary antibodies diluted 1:1000: anti-p27 (ab32034), anti-CDK4 (ab108357), anti-keratin 1 (ab185628), anti-keratin 5 (ab64081), anti-keratin 10 (ab237775), anti-filaggrin (sc-66192), anti-loricrin (ab137533), anti-p-STAT3 (phospho Y705) (ab76315), anti-cyclin D2 (ab207604), and anti-p-JAK1 (ab125051). Subsequently, the membranes were incubated with the corresponded secondary antibodies (1:5000) at room temperature for 1 h. Finally, the ECL kit (Thermo Fisher Scientific) was used to visualize the protein bands. Anti-filaggrin was provided by Santa Cruz Biotechnology, Inc. (Santa Cruz, CA, USA). Other antibodies were purchased from Abcam (Cambridge, MA, USA).

### Real-time quantitative PCR (RT-qPCR)

Total RNA was isolated from NHEK cells using Trizol reagent (ELK Biotechnology, Wuhan, China). EntiLink™ 1st Strand cDNA Synthesis Kit (ELK Biotechnology) was used for the synthesis of the first strand of cDNA. qPCR was performed on StepOne™ real-time PCR instrument (Life technologies) by using EnTurbo™ SYBR Green PCR SuperMix Kit (ELK Biotechnology). The following qPCR cycling conditions were used: 3 min 95° C, followed by 40 cycles of 10 s 95° C, 30 s 58° C, and 30 s 72° C. The primers are listed in Table 1. The 2^–ΔΔCT^ method was used for data analysis.

**Table 1:** Primer sequences.

**Name**		**Primer sequences (5’-3’)**
IL-6	Forward	ACTGGCAGAAAACAACCTGAAC
	Reverse	TTGTACTCATCTGCACAGCTCTG
IL-1β	Forward	ACGATGCACCTGTACGATCACT
	Reverse	GAGAACACCACTTGTTGCTCCA
CXCL1	Forward	AACCGAAGTCATAGCCACACTC
	Reverse	CTTCTCCTAAGCGATGCTCAAA
CXCL2	Forward	AAGGGGTTCGCCGTTCTC
	Reverse	TGGCAGCGCAGTTCAGTG
LL37	Forward	CTCAGCCTCTGCGGAGAAAG
	Reverse	CCACCTGAGCCCTATAAAAGATG
β- defensin2	Forward	ATGTCATCCAGTCTTTTGCCC
	Reverse	TGCGTATCTTTGGACACCATAG
S100A7	Forward	GCACAAATTACCTCGCCGAT
	Reverse	GACATTTTATTGTTCCTGGGGTC
S100A8	Forward	AAGTCCGTGGGCATCATGTT
	Reverse	TCAGGTCATCCCTGTAGACGG
CyclinD2	Forward	GGATGAGGAAGTGAGCTCGC
	Reverse	CTATTGAGGAGCACCGCCTC
β-actin	Forward	GTCCACCGCAAATGCTTCTA
	Reverse	TGCTGTCACCTTCACCGTTC

### ROS analysis

The Reactive Oxygen Species Assay Kit (Beyotime, Beijing, China) was used to detect the accumulation of intracellular ROS in NHEK cells. NHEK cells were stained with 2, 7-dichlorofluorescein diacetate staining solution at 37° C for 20 min. After that, the fluorescence intensity was analyzed by flow cytometry (BD Biosciences).

### ELISA assay

Concentrations of SOD, MDA, GSH and CAT in NHEK cell supernatants were measured using ELISA kits (Nanjing Jiancheng Bioengineering Institute (Nanjing, China)) according to manufacturer's protocols. Superoxide Dismutase (SOD) assay kit (A001-3), Malondialdehyde (MDA) assay kit (A003-1), Reduced glutathione (GSH) assay kit (A006-2) and Catalase (CAT) assay kit (A007-1) were obtained from Nanjing Jiancheng Bioengineering Institute (Nanjing, China).

### Statistical analysis

All statistical analyses were performed using the GraphPad Prism software (version 7.0, La Jolla, CA, USA). The comparison among multiple groups was analyzed with one-way analysis of variance (ANOVA) followed by Tukey’s test. All experiments were repeated three times. All data are shown as the mean ± SD; P<0.05 was considered as statistically significant.

### Availability of data and materials

The datasets used and/or analyzed in this study are available from the corresponding author upon a reasonable request.
